# Microcephaly-chorioretinopathy syndrome, autosomal recessive form. A case report: Síndrome de microcefalia-coriorretinopatia, forma autossômica recessiva. Um relato de caso

**DOI:** 10.1590/1516-3180.2013.7930003

**Published:** 2014-10-17

**Authors:** Rafael Fabiano Machado Rosa, Flávia Enk, Korine Camargo, Giovanni Marco Travi, André Freitas, Rosana Cardoso Manique Rosa, Carla Graziadio, Vinicius Freitas de Mattos, Paulo Ricardo Gazzola Zen

**Affiliations:** I PhD. Clinical Geneticist, Universidade Federal de Ciências da Saúde de Porto Alegre (UFCSPA) and Complexo Hospitalar Santa Casa de Porto Alegre (CHSCPA), Porto Alegre, Rio Grande do Sul, Brazil.; II Undergraduate Medical Student, Universidade Luterana do Brasil (ULBRA), Canoas, Rio Grande do Sul, Brazil.; III MD. Ophthalmologist, Complexo Hospitalar Santa Casa de Porto Alegre (CHSCPA), Porto Alegre, Rio Grande do Sul, Brazil.; IV MD. Pediatrician, Grupo Hospitalar Conceição (GHC), Porto Alegre, Rio Grande do Sul, Brazil.; V MD. Assistant Professor of Clinical Genetics and Student in the Postgraduate Program on Pathology, Universidade Federal de Ciências da Saúde de Porto Alegre (UFCSPA), and Clinical Geneticist, Universidade Federal de Ciências da Saúde de Porto Alegre (UFCSPA) and Complexo Hospitalar Santa Casa de Porto Alegre (CHSCPA), Porto Alegre, Rio Grande do Sul, Brazil.; VI MD. Clinical Geneticist, Universidade Federal de Ciências da Saúde de Porto Alegre (UFCSPA) and Complexo Hospitalar Santa Casa de Porto Alegre (CHSCPA), Porto Alegre, Rio Grande do Sul, Brazil.; VII PhD. Adjunct Professor of Clinical Genetics and of the Postgraduate Program on Pathology, Universidade Federal de Ciências da Saúde de Porto Alegre (UFCSPA), and Clinical Geneticist, Universidade Federal de Ciências da Saúde de Porto Alegre (UFCSPA) and Complexo Hospitalar Santa Casa de Porto Alegre (CHSCPA), Porto Alegre, Rio Grande do Sul, Brazil.

**Keywords:** Microcephaly, Retina, Intellectual disability, Consanguinity, Toxoplasmosis, Microcefalia, Retina, Deficiência intelectual, Consanguinidade, Toxoplasmose

## Abstract

**CONTEXT:**

The autosomal recessive form of microcephaly-chorioretinopathy syndrome is a rare genetic condition that is considered to be an important differential diagnosis with congenital toxoplasmosis.

**CASE REPORT:**

Our patient was a seven-year-old white boy who was initially diagnosed with congenital toxoplasmosis. However, his serological tests for congenital infections, including toxoplasmosis, were negative. He was the first child of young, healthy and consanguineous parents (fourth-degree relatives). The parents had normal head circumferences and intelligence. The patient presented microcephaly and specific abnormalities of the retina, with multiple diffuse oval areas of pigmentation and patches of chorioretinal atrophy associated with diffuse pigmentation of the fundus. Ophthalmological evaluations on the parents were normal. A computed tomography scan of the child’s head showed slight dilation of lateral ventricles and basal cisterns without evidence of calcifications. We did not find any lymphedema in his hands and feet. He had postnatal growth retardation, severe mental retardation and cerebral palsy.

**CONCLUSIONS:**

The finding of chorioretinal lesions in a child with microcephaly should raise suspicions of the autosomal recessive form of microcephaly-chorioretinopathy syndrome, especially in cases with an atypical pattern of eye fundus and consanguinity. A specific diagnosis is essential for an appropriate clinical evaluation and for genetic counseling for the patients and their families.

## RESUMO

**CONTEXTO:**

A forma autossômica recessiva da síndrome de microcefalia-coriorretinopatia é condição genética rara, considerada um importante diagnóstico diferencial com toxoplasmose congênita.

**RELATO DO CASO:**

O paciente era um menino branco de sete anos de idade, inicialmente diagnosticado com toxoplasmose congênita. No entanto, suas sorologias para infecções congênitas, incluindo a toxoplasmose, eram negativas. Ele foi o primeiro filho de pais jovens, hígidos e consanguíneos (parentes de quarto grau). Os pais apresentavam perímetro cefálico e inteligência normais. O paciente apresentava microcefalia e anormalidades específicas da retina com áreas ovais de pigmentação múltiplas e difusas, além de manchas de atrofia coriorretiniana associadas à pigmentação difusa do fundo de olho. A avaliação oftalmológica dos pais foi normal. A tomografia computadorizada de crânio da criança mostrou discreta dilatação dos ventrículos laterais e cisternas basais, sem evidência de calcificações. Nós não verificamos a presença de linfedema em suas mãos e pés. Ele possuía retardo do crescimento pós-natal, deficiência mental grave e paralisia cerebral.

**CONCLUSÃO:**

O achado de lesões coriorretinianas em uma criança com microcefalia deve aumentar a suspeita da forma autossômica recessiva da síndrome de microcefalia-coriorretinopatia, principalmente em casos com padrão atípico de fundo de olho e consanguinidade. O diagnóstico preciso é essencial para correta avaliação clínica e aconselhamento genético dos pacientes e suas famílias.

## INTRODUCTION

The findings of microcephaly and chorioretinopathy in a newborn usually lead to the hypothesis of congenital infection, especially in countries where some of these diseases, like toxoplasmosis, are prevalent.^1^ However, these features have been described in families presenting both autosomal dominant and recessive patterns of inheritance.^2^^,^^3^^,^^4^

The aim of our report was to describe a boy who presented microcephaly-chorioretinopathy syndrome that was compatible with an autosomal recessive form. This is a rare condition that is considered to be an important differential diagnosis with congenital toxoplasmosis.

## CASE REPORT

**Figure 1.**
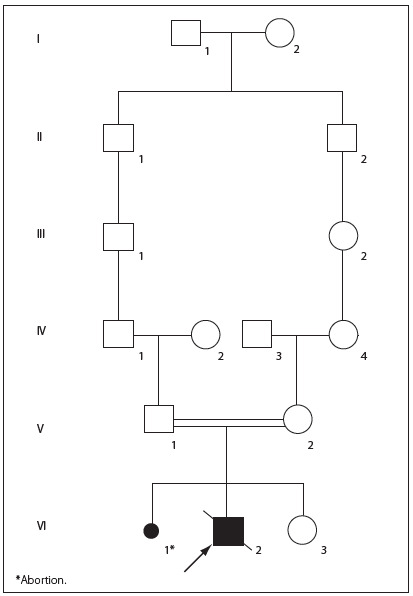
Pedigree of the family showing the consanguinity observed between the patient’s parents.

Our patient was a seven-year-old white boy who was initially diagnosed with congenital toxoplasmosis. He was the first child of young, healthy and consanguineous parents (fourth-degree relatives), and had a healthy sister of three years of age (Figure 1). The mother had a history of one previous loss of pregnancy. She said that she had not smoked, consumed alcohol or made use of illicit drugs during the pregnancy. The family history was negative for similar cases. The parents had normal head circumferences and intelligence. The child was born from an uneventful pregnancy, by means of cesarean delivery, at eight months of gestational age, weighing 2,740 g (i.e. within the range of the 50-90^th^ percentiles), measuring 47 cm (50-98^th^ percentiles), with head circumference of 32 cm (10-50^th^ percentiles) and Apgar score of 9 at five minutes. Serological tests for congenital infections (toxoplasmosis, rubella, cytomegalovirus, herpes simplex and syphilis) were negative. No lymphedema was observed in his hands and feet and the patient also did not present anemia, petechiae, maculopapular rash or jaundice.

**Figure 2.**
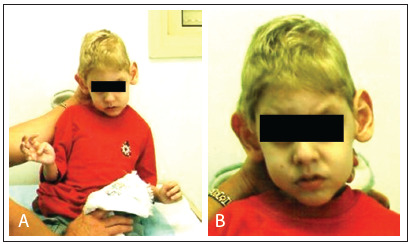
Appearance of the patient at five years of age showing microcephaly, prominent large ears, pointed chin (A and B), and spasticity of upper limbs (A).

He was hospitalized due to pneumonia on four occasions, the first at four months of age. At four years and seven months, his weight was 11 kg (< 3^rd^ percentile), length 101 cm (< 3^rd^ percentile), head circumference 42 cm (< 2^nd^ percentile) and ear length 6.5 cm (> 97^th^ percentile). He had a high arched palate, prominent large ears, pointed chin, spasticity and atrophy of the upper and lower limbs, right-side cryptorchid testis and bilateral overlapping of the second and fourth toes over the third toes (Figure 2). In the neurological evaluation, he was hypertonic, presented little social interaction and had significant neuropsychomotor delay. He was not capable of supporting his head or speaking words, but he did not have seizures. A computed tomography scan of the head showed slight dilation of lateral ventricles and basal cisterns without evidence of calcifications. Electroencephalographic evaluation showed a cerebral pattern with little organization and subcortical paroxysms.

**Figure 3.**
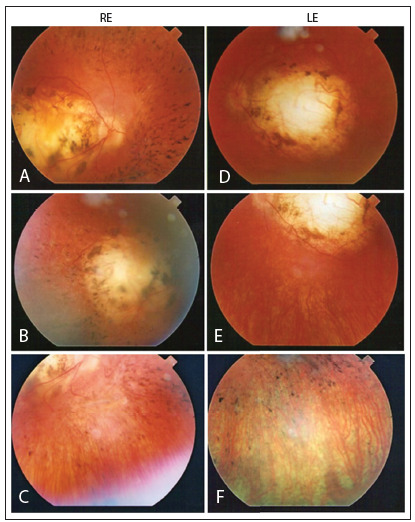
Images of the eye fundus examination showing peripapillary retinal atrophy; abnormality of the peripheral retinal pigment epithelium, typical of chorioretinopathy, with poorly defined borders and little perilesional pigmentation; and a minor juxtapapillary lesion occupying the macula and multiple clumps of pigment spread across the retina in the right eye (RE: right eye; LE: left eye).

In an eye examination, he did not fix on or follow objects and he was unable to perform the Snellen visual acuity test. He did not have any relative afferent pupillary defect, ocular misalignment or abnormalities in the slit-lamp examination. Significant blepharitis was observed in both eyes. His pupils were isochoric and, in an eye fundus examination, peripapillary retinal atrophy was observed. There was abnormality of the peripheral retinal pigment epithelium, typical of chorioretinopathy, with poorly defined borders and little perilesional pigmentation, along with a minor juxtapapillary lesion occupying the macula and multiple clumps of pigment spread across the retina (Figure 3). Ophthalmological assessments on his parents and sister were normal.

The radiological investigation showed microcephaly and bilateral hip dislocation. High resolution GTG-banded karyotyping was normal (46,XY). He developed chickenpox and died as a result of complications at 11 years of age, and no electroretinography could be performed at that time. No autopsy was performed.

## DISCUSSION

Our patient presented microcephaly and specific abnormalities of the retina with multiple diffuse oval areas of pigmentation and patches of chorioretinal atrophy associated with diffuse pigmentation of the fundus, and a family history of consanguinity between the parents. The ophthalmological evaluations on these first-degree relatives were normal. Our patient, similar to those described by Schmidt et al.^3^ and Abdel-Salam et al.,^5^ also presented postnatal growth retardation, severe mental retardation and cerebral palsy. These findings are consistent with the autosomal recessive form of microcephaly-chorioretinopathy syndrome (OMIM #251270).^6^ Lymphedema, a feature not seen in our patient, has also been described only in association with families presenting dominant inheritance.^7^

In our review of the literature, using the descriptors “Microcephaly” AND “Chorioretinopathy” AND “(Autosomal Recessive)”, we found only two related articles (one case report and one original article) (Table 1).^4^^,^^8^ The case report was made by Cantú et al.^4^ The authors described two sisters and their brother who presented microcephaly, microphthalmia, chorioretinal degeneration and optic atrophy. Similar to our patient, they also had delayed growth and development.^4^ Consanguinity, a feature seen in our family, was also suspected by Cantú et al.,^4^ because the parents were born in the same village and two of their grandparents had the same unusual last name. Nonetheless, the distribution of the affected individuals (two sisters and their brother, with unaffected parents) suggests an autosomal recessive pattern of inheritance. The recessive form of microcephaly-chorioretinopathy syndrome is considered to be a very rare condition and has been correlated with homozygous mutations in the *TUBGCP6* gene on chromosome 22q.^8^

**Table 1.**
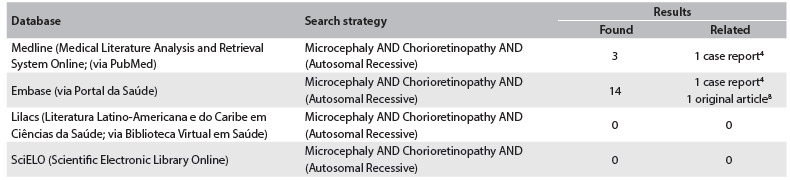
Results obtained from each database using the descriptor of the diagnosis presented by the patient. The search in these databases was conducted on November 29, 2013

The finding of retinal lesions in a child with microcephaly suggests the diagnosis of congenital toxoplasmosis, especially in endemic areas such as Brazil, as observed with our patient.^1^ If a pregnant woman acquires primary infection, *Toxoplasma gondii* may be transmitted to the fetus and cause inflammatory lesions that may lead to permanent neurological damage, including microcephaly and chorioretinopathy.^1^ The chorioretinopathy of microcephaly-chorioretinopathy syndrome is reminiscent of that associated with congenital toxoplasmosis. Because of the similarity of the findings, some authors have suggested the designation “pseudotoxoplasmosis” for this syndrome.^9^ However, the chorioretinopathy of toxoplasmosis is more confined to the perimacular region and is more associated with other eye abnormalities such as microphthalmia and cataracts, as well as intracranial calcifications and seizures.^10^ Thus, the chorioretinal changes present in patients with microcephaly-chorioretinopathy syndrome differ from the scars of toxoplasmic chorioretinopathy because of their multiplicity and widespread localization, as observed in our patient. The finding in our patient of absence of immunoglobulin M (IgM)-specific antibodies for toxoplasmosis also help to rule out the possibility of diagnosing this congenital infection.

## CONCLUSIONS

Thus, the finding of chorioretinal lesions in a child with microcephaly should also raise suspicions of the autosomal recessive form of microcephaly-chorioretinopathy syndrome, especially in cases with an atypical pattern of eye fundus and family history of consanguinity. This is essential for an appropriate clinical evaluation and for genetic counseling for the patients and their families.

## References

[1] Petersen E (2007). Toxoplasmosis. Semin Fetal Neonatal Med.

[2] McKusick VA, Stauffer M, Knox L, Clark DB (1966). Chorioretinopathy with hereditary microcephaly. Arch Ophthalmol.

[3] Schmidt B, Jaeger W, Neubauer H (1967). Ein Mikrozephalie-Syndrom mit atypischer tapetoretinaler degeneration bei 3 Geschwistern [A microcephalic syndrome with atypical tapetoretinal degeneration in 3 siblings]. Klin Monbl Augenheilkd.

[4] Cantú JM, Rojas JA, García-Cruz D (1977). Autosomal recessive microcephaly associated with chorioretinopathy. Hum Genet.

[5] Abdel-Salam GM, Czeizel AE, Vogt G, Imre L (2000). Microcephaly with chorioretinal dysplasia: characteristic facial features. Am J Med Genet.

[6] Microcephaly and chorioretinopathy with or without mental retardation, autosomal recessive. OMIM^®^. Online Mendelian Inheritance in Man^®^.

[7] Casteels I, Devriendt K, Van Cleynenbreugel H (2001). Autosomal dominant microcephaly--lymphoedema-chorioretinal dysplasia syndrome. Br J Ophthalmol.

[8] Puffenberger EG, Jinks RN, Sougnez C (2012). Genetic mapping and exome sequencing identify variants associated with five novel diseases. PLoS One.

[9] McKusick VA (1994). Mendelian Inheritance in Man.

[10] Kodjikian L, Wallon M, Fleury J (2006). Ocular manifestations in congenital toxoplasmosis. Graefes Arch Clin Exp Ophthalmol.

